# Response Rate Patterns in Adolescents With Concussion Using Mobile Health and Remote Patient Monitoring: Observational Study

**DOI:** 10.2196/53186

**Published:** 2024-05-06

**Authors:** Sicong Ren, Catherine C McDonald, Daniel J Corwin, Douglas J Wiebe, Christina L Master, Kristy B Arbogast

**Affiliations:** 1 Center for Injury Research and Prevention, The Children’s Hospital of Philadelphia, Philadelphia, PA, United States; 2 Perelman School of Medicine, University of Pennsylvania, Philadelphia, PA, United States; 3 School of Nursing, University of Pennsylvania, Philadelphia, PA, United States; 4 Division of Emergency Medicine, The Children’s Hospital of Philadelphia, Philadelphia, PA, United States; 5 Department of Emergency Medicine, University of Michigan, Ann Arbor, MI, United States; 6 Department of Epidemiology, School of Public Health, University of Michigan, Ann Arbor, PA, United States; 7 Sports Medicine and Performance Center, The Children’s Hospital of Philadelphia, Philadelphia, PA, United States

**Keywords:** concussion, mHealth, response rate, adolescents, reporting behavior, remote monitoring, engagement, monitoring, adolescent, teen, youth, remote patient monitoring, mobile health, injury, neurobiological, neurobiological development, quality of life, academic, academic performance, mHealth engagement, tertiary care

## Abstract

**Background:**

A concussion is a common adolescent injury that can result in a constellation of symptoms, negatively affecting academic performance, neurobiological development, and quality of life. Mobile health (mHealth) technologies, such as apps for patients to report symptoms or wearables to measure physiological metrics like heart rate, have been shown to be promising in health maintenance. However, there is limited evidence about mHealth engagement in adolescents with a concussion during their recovery course.

**Objective:**

This study aims to determine the response rate and response rate patterns in concussed adolescents reporting their daily symptoms through mHealth technology. It will also examine the effect of time-, demographic-, and injury-related characteristics on response rate patterns.

**Methods:**

Participants aged between 11-18 years (median days since injury at enrollment: 11 days) were recruited from the concussion program of a tertiary care academic medical center and a suburban school’s athletic teams. They were asked to report their daily symptoms using a mobile app. Participants were prompted to complete the Post-Concussion Symptom Inventory (PCSI) 3 times (ie, morning, afternoon, and evening) per day for 4 weeks following enrollment. The primary outcome was the response rate pattern over time (by day since initial app use and the day since injury). Time-, demographic-, and injury-related differences in reporting behaviors were compared using Mann Whitney *U* tests.

**Results:**

A total of 56 participants were enrolled (mean age 15.3, SD 1.9 years; n=32, 57% female). The median response rate across all days of app use in the evening was 37.0% (IQR 27.2%-46.4%), which was significantly higher than the morning (21.2%, IQR 15.6%-30.5%) or afternoon (26.4%, IQR 21.1%-31.5%; *P*<.001). The median daily response was significantly different by sex (female: 53.8%, IQR 46.2%-64.2% vs male: 42.0%, IQR 28.6%-51.1%; *P*=.003), days since injury to app use (participants starting to use the app >7 days since injury: 54.1%, IQR 47.4%-62.2% vs starting to use the app ≤7 days since injury: 38.0%, IQR 26.0%-53.3%; *P*=.002), and concussion history (participants with a history of at least one prior concussion: 57.4%, IQR 44.5%-70.5% vs participants without concussion history: 42.3%, IQR 36.8%-53.5%; *P*=.03). There were no significant differences by age. Differences by injury mechanism (sports- and recreation-related injury: 39.6%, IQR 36.1%-50.4% vs non–sports- or recreation-related injury: 30.6%, IQR 20.0%-42.9%; *P*=.04) and initial symptom burden (PCSI scores greater than the median score of 47: 40.9%, IQR 35.2%-53.8% vs PCSI scores less than or equal to the median score: 31.9%, IQR 24.6%-40.6%; *P*=.04) were evident in the evening response rates; however, daily rates were not statistically different.

**Conclusions:**

Evening may be the optimal time to prompt for daily concussion symptom assessment among concussed adolescents compared with morning or afternoon. Multiple demographic- and injury-related characteristics were associated with higher daily response rates, including for female participants, those with more than 1 week from injury to beginning mHealth monitoring, and those with a history of at least one previous concussion. Future studies may consider incentive strategies or adaptive digital concussion assessments to increase response rates in populations with low engagement.

## Introduction

Concussion is a significant public health problem that affects approximately 20% of adolescents in the United States each year [1,2]. The prevalence of lifetime concussions has been estimated to be between 6.5% and 18.3% among adolescents aged 13-17 years [3], with approximately 13.7% experiencing persistent symptoms for at least 3 months [4]. Given the critical developmental changes that occur during this period, persistent symptom burden and delayed recovery from concussion may negatively affect adolescents’ neurobiological and cognitive development [5].

Adolescents with a concussion can present with a variable symptom burden and recovery trajectories. Prior studies have shown that sex, age, nonadherence to medical care, injury mechanism, and previous history of concussion have been associated with prolonged recovery times [6-9]. For example, patients less likely to adhere to follow-up recommendations had a higher risk of prolonged recovery than those who demonstrated consistent medical adherence [9]. Given the variability in symptom burden and recovery, concussion evaluation, treatment, and follow-up recommendations must be individualized for each adolescent [10]. For instance, based on an individual’s symptom constellation, treatments could be tailored into multiple lines such as cognition, vision, and behavioral therapies [10-12]. Individualized treatment relies on the real-time monitoring of symptoms in natural environments [13].

The adoption of ecological momentary assessments using mobile health (mHealth) has increased for monitoring disease prognosis and rehabilitation [14,15], and can be used in concussion symptom monitoring. mHealth apps have been developed for patients with a concussion to report their real-time symptoms and activities multiple times per day to collect more granular information on symptom trajectory than is provided during periodic clinic visits [16,17].

Previous studies have used mHealth technology to facilitate follow-up for patients with traumatic brain injury, providing an opportunity for patients to report important details on the variability of symptoms and other sequelae [14,18]. Patients can provide real-time symptoms instead of relying on memory at the next in-person visit, which can be influenced by recall bias [19]. Documenting symptom presence and intensity in the moment can improve the reliability of self-report data and augment care individualization. While these studies show promise for these mHealth technologies, understanding the parameters that influence a patient’s engagement with the technology is critical to optimizing future implementation. Adolescents represent a unique patient population in this regard, as they are both high users of mobile technology while at the same time generally less engaged health care consumers [20]. In a systematic review, Wen et al [15] summarized nearly 20 studies that used mHealth technology to engage youth from a variety of clinical settings, reporting that patients on average responded to 77% of the prompts. These studies examined a wide range of patient behaviors across several diseases. Specific to concussion, several studies examined mHealth technologies to track symptoms or activity after injury. Reporting behaviors (eg, response rate) ranged from 50% to 90% due to differing assessments, tracking frequencies, and duration of follow-up [15-17,21,22]. Furthermore, these studies tend to reduce response rates to a single number (eg, the percentage of days in which participants completed responses divided by the number of prompts received [15,23]), with limited exploration of variability in response patterns within a day, across recovery duration, or by certain patient or injury characteristics. Understanding trends and factors influencing adolescent reporting behaviors is important to assist both clinicians and researchers in designing mHealth strategies to investigate concussion recovery trajectories and provide individualized management strategies.

The objectives of this study were to determine the response rate pattern over time to prompts for reporting daily symptoms in concussed adolescents through mHealth technology; identify the time of day when response rates were the highest; and examine the effect of time-, demographic-, and injury-related characteristics on response rate patterns.

## Methods

### Ethical Considerations

This study was approved by the Children’s Hospital of Philadelphia institutional review board (17-013875 and 18-014862). Participants or their parents or legal guardians provided verbal assent or written informed consent. Of note, while participants received a financial incentive to participate in the overall study, they did not receive additional compensation to respond to the mHealth prompts. Data were deidentified.

### Study Design and Participants

This study was a prospective observational cohort study approved by our institution’s review board. Participants with a concussion aged 11-18 years were recruited between September 28, 2018, and June 8, 2021, from the concussion clinic of our tertiary care academic medical center and a suburban school’s athletic teams as part of a larger prospective observational study [24]. The diagnosis of concussion was made by a trained sports medicine pediatrician following the most recent international consensus statement on concussion [25]. All participants had an initial in-person clinical assessment, either as part of a clinic visit or in the school’s athletic training room, within 28 days of injury. Enrollment in the mHealth study could occur at this initial visit or a subsequent visit. Participants were excluded from enrollment in the larger study if they were still recovering from a previous concussion (or within 30 days of clearance from a previous concussion). Participants were excluded from this analysis if there were missing demographic or injury variables. Injury mechanism and concussion history were not recorded for 1 participant, who was thus excluded from specific subgroup analyses.

Upon enrollment, participants were instructed to download the mHealth tool onto their smartphones via an SMS text message invitation. This tool allowed the participants to report their symptoms 3 times daily for 28 days following enrollment. The details of the mHealth tool are described below.

For analyses, participant age, sex, date of injury, injury mechanism, and concussion history were abstracted from the electronic medical record.

### mHealth Tool

Recovering Concussion Update on the Progression of Symptoms (ReCoUPS) is a mobile app protocol developed by the study team for patients to report real-time symptoms and activities following concussion. The ReCoUPS app “pings” (prompting with a chime or vibration) participants 3 times per day: morning (9 AM to 1 PM), afternoon (4-7 PM), and evening (8 PM). Participants reported symptoms using the Post-Concussion Symptom Inventory (PCSI) [26]. The adolescent PCSI (used for participants aged ≥13 years) includes 21 concussion symptoms rated using a 7-point Likert scale from 0 (none) to 6 (severe), with total symptom scores ranging from 0 to 21 and total symptom severity scores ranging from 0 to 126. The child PCSI (used for younger children aged <13 years) is a 17-item symptom checklist with symptoms rated on a 3-point scale from 0 (none) to 2 (a lot), with total symptom scores ranging from 0 to 17 and total symptom severity scores ranging from 0 to 34. The PCSI has been demonstrated to be a valid and reliable symptom assessment in the pediatric setting [26].

### Data Analysis

The response rate was computed through 2 time-based approaches: (1) response rate by days since first app use and (2) response rate by days since injury. The two response rates examine response trends over time, examining the median response across days following injury. These 2 time-based response rates differ from one another as patients enrolled in the app during regular clinical visits that occurred at varying times since injury.

Specifically, response rate by days since first app use and response rate by days since injury were calculated as the number of completed prompts on a given day divided by the number of prompts received on that day across all participants for each day since first app use and each day since injury, respectively. Response rate was examined both by session time (morning, afternoon, and evening prompts; equation 1 in Multimedia Appendix 1) and daily (responding at least once per day, equation 2 in Multimedia Appendix 1). Thus, response rate by day represents the percentage of participants who completed prompts each session time (or at least once daily) on each day (ie, single value per day and time series for response rate trend).

Additionally, subgroup response rates were computed. Subgroups included demographics (sex and age), injury features (injury mechanism, concussion history, initial symptom burden on enrollment), and time between the date of injury and date of first app use (ie, days from injury to app use). The age range was categorized as younger teens (ie, ages 11-14 y) and older teens (ages 15-18 y). Days from injury to app use was categorized into two groups: ≤7 days and >7 days. Initial symptom burden was categorized into two groups based on the median initial PCSI score of the overall sample: ≤47.0 points and >47.0 points. Since there were only complete initial PCSI scores for children aged 13-18 years, this scale was relevant for all participants with valid data.

Descriptive statistics were used to summarize demographic and injury characteristics. For continuous variables, means with SDs were computed for normally distributed data, and medians with IQRs were computed for skewed data. For categorical variables, frequencies were computed. For each type of response rate, the Friedman test was used to examine the differences in response rate based on session time (ie, morning, afternoon, and evening). Post hoc analyses were performed via pairwise Mann Whitney *U* test with Bonferroni *P* value adjustment. Differences in response rate by days since first app use were tested across demographic-related (ie, sex and age), injury-related (ie, injury mechanism, concussion history, and initial symptom burden on enrollment), and days from injury to app use subgroups through Mann Whitney *U* tests. Comparisons of response rate by days since injury based on sex, age, injury mechanism, concussion history, and initial symptom burden were performed using Mann Whitney *U* test. Differences in the initial symptom burden at the clinical visit by all subgroups were also tested via Mann Whitney *U* test. All statistical analyses were performed using R version 4.2.2 (R Foundation for Statistical Computing). The significance level was set at .05.

## Results

### Sample Composition

**Figure 1.**
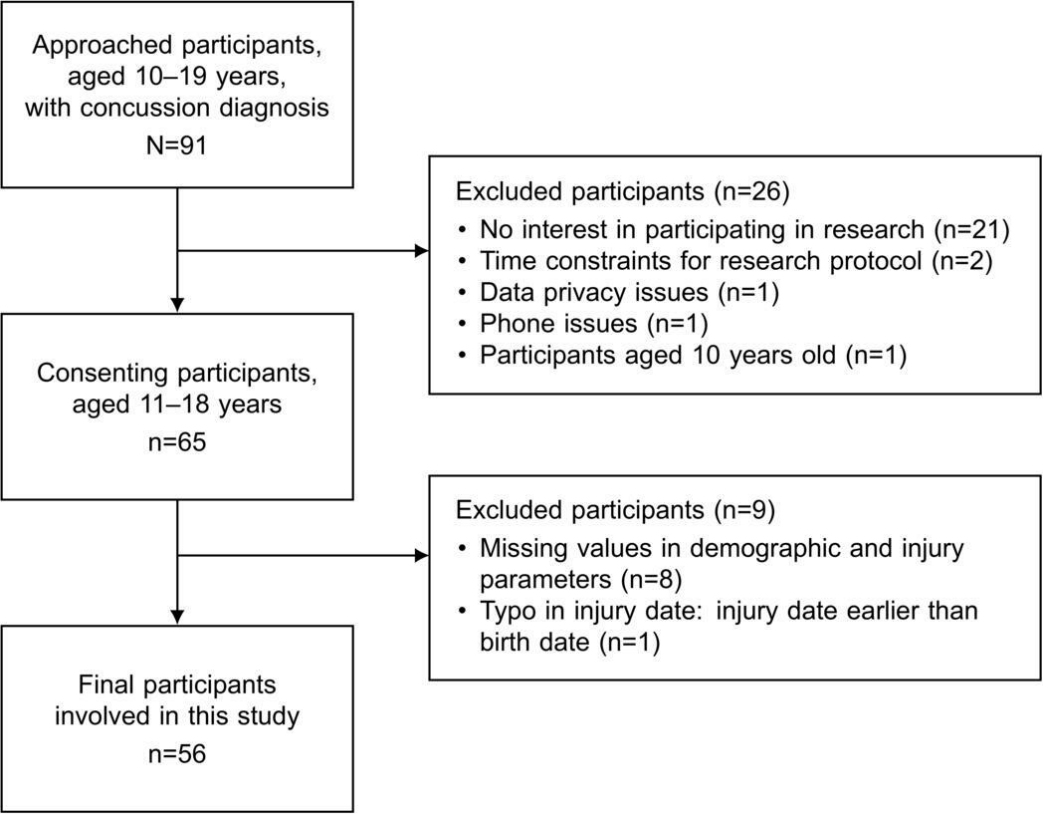
Flowchart of participant selection in this study.

The final sample included 24 (43%) male participants and 32 (57%) female participants with an average age of 15.3 (SD 1.9) years. The flowchart of participant inclusion is shown in Figure 1. Table 1 summarizes the demographic and injury characteristics of the study cohort. The final injury-related subgroups included 34 (61%) participants with sports- and recreation-related injuries and 21 (38%) with non–sports- and recreation-related injuries. There were 30 (54%) participants with a history of at least one prior concussion and 25 (45%) without concussion history. No significant differences in the initial symptom burden at clinic visits by demographic- and injury-related groups were observed.

**Table 1.** Demographic and injury characteristics of participants (N=56).

		Value
**Sex, n (%)**		
	Female	32 (57)
	Male	24 (43)
**Race/ethnicity, n (%)**		
	Non-Hispanic White	40 (71)
	Non-Hispanic Black	2 (4)
	Hispanic	4 (7)
	Other	10 (18)
**Mechanism of injury, n (%)**		
	Sports- and recreation-related injury	34 (61)
	Non–sports- and recreation-related injury	21 (37)
	Not reported	1 (2)
**Previous history of concussion, n (%)**		
	Yes	30 (53)
	No	25 (45)
	Not reported	1 (2)
Age at injury (years), median (IQR)		15.0 (14.0-17.0)
Post-Concussion Symptom Inventory total score at initial clinic visit, median (IQR)		47.0 (27-68)
Days since injury at enrollment, median (IQR)		11.0 (6-21)

### Overall Response Rate Pattern

**Figure 2.**
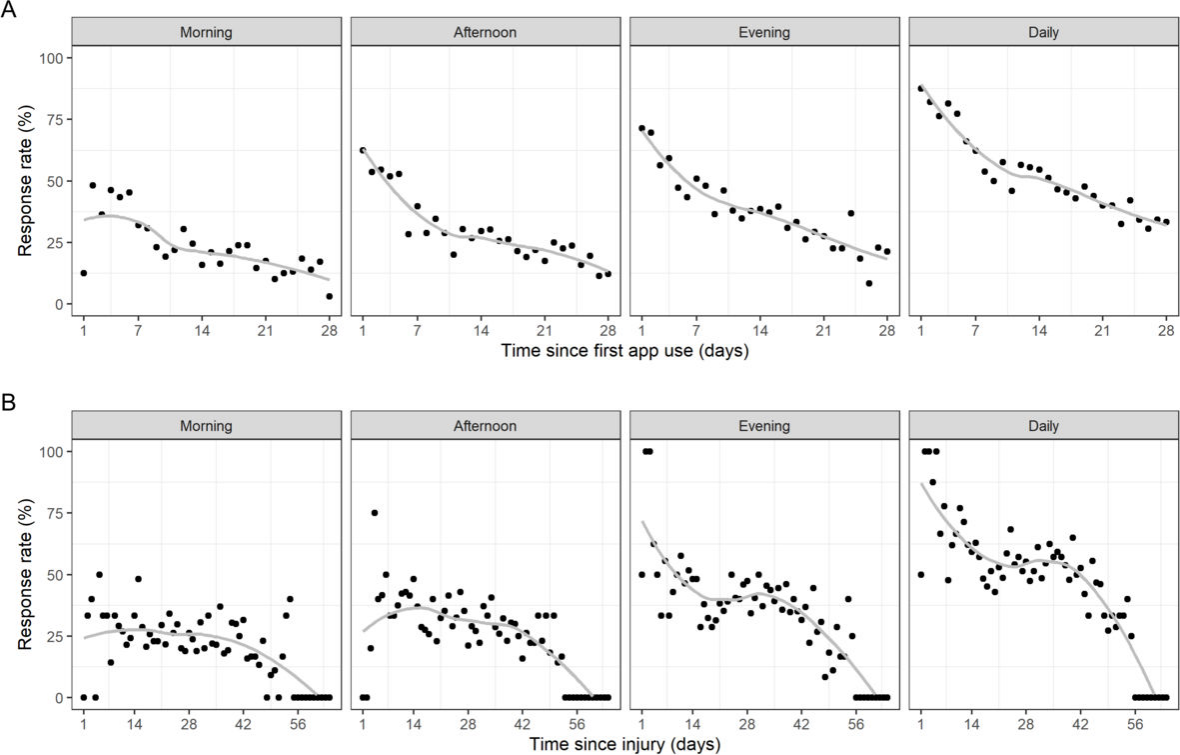
Response rate pattern by morning, afternoon, and evening, and daily by (A) days since first app use and (B) days since injury. Each black dot indicates the response rate that day, defined as the number of participants who completed prompts divided by the number of participants who received notifications for that day. The solid gray lines represent smoothed trend lines through local regression. A daily response was defined as a participant completing a response at least once per day (morning, afternoon, or evening).

The response pattern varied substantially over time. On average, response rates by days since first app use started high (49/56, 88% of participants responded at least once on the first day of using the app) and decreased by approximately 50% over the duration of the study, with the steepest declines within the first 10 days (Figure 2, top). When considering variations by session time, the evening response rate was generally higher than the morning and afternoon response rates. The daily response rate by days since injury also started high, declined to approximately 50% (18/34) at day 21, plateaued until day 35, and then decreased sharply until the completion of the study period (Figure 2, bottom). The plateau reflects the varying times post injury that participants enrolled in the study (Figure S1 in Multimedia Appendix 1). For example, 23% (15/56) of the participants started using the app between days 21 and 28 post injury.

### Response Rates by Days Since First App Use

**Figure 3.**
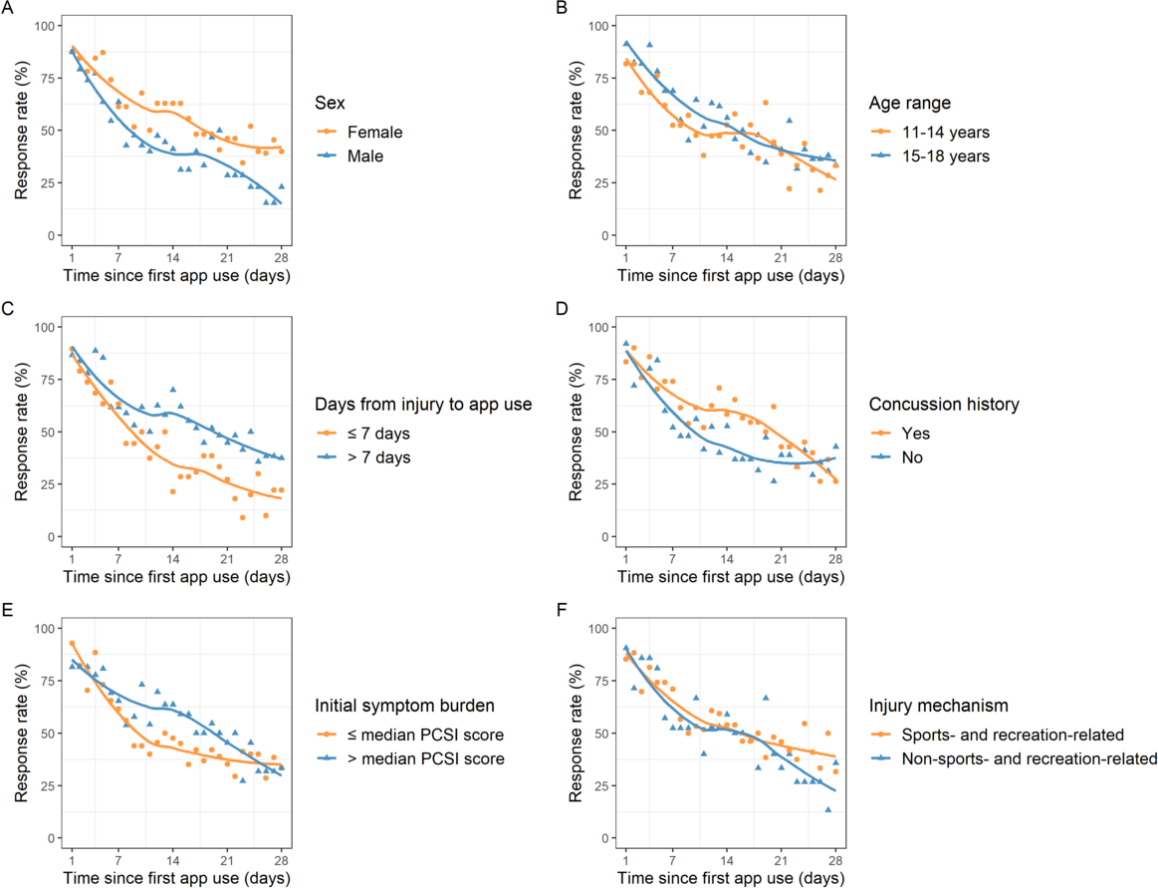
Comparisons of daily response rate by days since first app use by (A) sex, (B) age group, (C) days from injury to app use, (D) concussion history, (E) initial symptom burden, and (F) injury mechanism. The solid lines represent smoothed trend lines through local regression. Daily response was defined as a participant completing a response at least once per day (morning, afternoon, or evening). Response rate was defined as the number of participants who completed prompts divided by the number of participants who received notifications for that day. PCSI: Post-Concussion Symptom Inventory.

Figure 3 and Table 2 show comparisons of response rate patterns by days since first app use for time-, demographic-, and injury-related groups. Overall, the evening response (median 37.0%, IQR 27.2%-46.4%) was significantly higher than the morning response (median 21.2%, IQR 15.6%-30.5%; *P*<.001). For demographic-related comparisons, many of the differences were driven by differences in the evening response rates. For female patients with concussion, evening (median 44.4%, IQR 33.0%-52.6% vs median 25.0%, IQR 14.3%-34.1%; *P*<.001) and daily response rates (median 53.8%, IQR 46.2%-64.2% vs median 42.0%, IQR 28.6%-51.1%; *P*=.003) were significantly higher than those in male patients with concussion. There were no significant differences by age. For injury-related comparisons, afternoon (median 30.4%, IQR 24.7%-39.0% vs median 24.3%, IQR 14.0%-30.9%; *P*=.04) and evening response rates (median 39.6%, IQR 36.1%-50.4% vs 30.6%, IQR 20.0%-42.9%; *P*=.04) in the sports- and recreation-related injury group were significantly higher than those in the non–sports- and recreation-related injury group; however, daily rates were not statistically different. Participants with a history of at least one prior concussion had significantly higher responses rates at every session time than those without injury history (eg, median daily response rate: 57.4%, IQR 44.5%-70.5% vs 42.3%, IQR 36.8%-53.5%; *P*=.03). Participants who started to use the app after 7 days post injury demonstrated significantly higher morning (median 23.8%, IQR 17.2%-34.6% vs median 14.3%, IQR 8.9%-23.9%; *P*=.006), evening (median 42.1%, IQR 31.0%-53.0% vs median 22.6%, IQR 16.4%-38.2%; *P*=.002), and daily responses rates (median 54.1%, IQR 47.4%-62.2% vs median 38.0%, IQR 26.0%-53.3%; *P*=.002) than those who started to use the app sooner. Participants whose initial symptom burden was larger than the median PCSI score showed higher evening response rates than those whose burden was smaller than or equal to the median PCSI score (median 40.9%, IQR 35.2%-53.8% vs median 31.9%, IQR 24.6%-40.6%; *P*=.04); however, daily rates were not statistically different.

**Table 2.** Response rate by days since first app use overall and by time-, demographic-, and injury-related features. The value in the table represents the median response rate across the reporting days and is complementary to the time trends in Figure 3.

Variables		Morning (%)		Afternoon (%)		Evening (%)		Daily (%)	
		Median IQR	*P* value	Median IQR	*P* value	Median IQR	*P* value	Median IQR	*P* value
Overall		21.2 (15.6-30.5)	—^a^	26.4 (21.1-31.5)	—	37.0 (27.2-46.4)	—	48.8 (41.6-58.8)	—
**Days from injury to app use**			.006		.05		.002		.002
	≤7 days (n=19)	14.3 (8.9-23.9)		22.6 (13.5-30.4)		22.6 (16.4-38.2)		38.0 (26.0-53.3)	
	>7 days (n=36)	23.8 (17.2-34.6)		29.8 (20.7-39.0)		42.1 (31.0-53.0)		54.1 (47.4-62.2)	
**Sex**			.07		.05		<.001		.003
	Male (n=24)	16.7 (7.7-29.4)		21.2 (15.1-33.9)		25.0 (14.3-34.1)		42.0 (28.6-51.1)	
	Female (n=32)	22.6 (18.2-32.3)		31.4 (22.9-38.0)		44.4 (33.0-52.6)		53.8 (46.2-64.2)	
**Age group (years)**			.58		.81		.40		.34
	11-14 (n=22)	21.1 (13.4-24.4)		27.0 (18.5-39.3)		36.8 (26.0-42.9)		47.5 (37.8-58.9)	
	15-18 (n=34)	21.7 (13.6-36.8)		26.7 (22.1-30.9)		39.4 (28.2-47.3)		50.9 (40.5-65.6)	
**Injury mechanism**			.05		.04		.04		.31
	SRR^b^ injury (n=34)	23.1 (20.8-35.5)		30.4 (24.7-39.0)		39.6 (36.1-50.4)		52.5 (46.1-63.0)	
	Non-SRR injury (n=21)	19.0 (6.7-27.1)		24.3 (14.0-30.9)		30.6 (20.0-42.9)		51.2 (35.1-60.8)	
**Concussion history**			.01		.01		.02		.03
	Yes (n=30)	22.9 (19.0-36.5)		33.3 (23.3-42.6)		43.2 (33.0-50.5)		57.4 (44.5-70.5)	
	No (n=25)	12.2 (10.5-26.7)		21.6 (16.4-28.0)		31.6 (21.9-44.0)		42.3 (36.8-53.5)	
**Initial symptom burden**			.35		.69		.04		.06
	Score >47 (median score; n=27)	19.5 (13.6-30.8)		28.2 (21.9-36.5)		40.9 (35.2-53.8)		56.1 (48.9-69.3)	
	Score ≤47 (median score; n=25)	23.3 (16.4-32.0)		24.5 (17.6-37.2)		31.9 (24.6-40.6)		43.1 (38.8-57.4)	

a Not applicable.

b SRR: sports- and recreation-related.

### Response Rates by Days Since Injury

**Figure 4.**
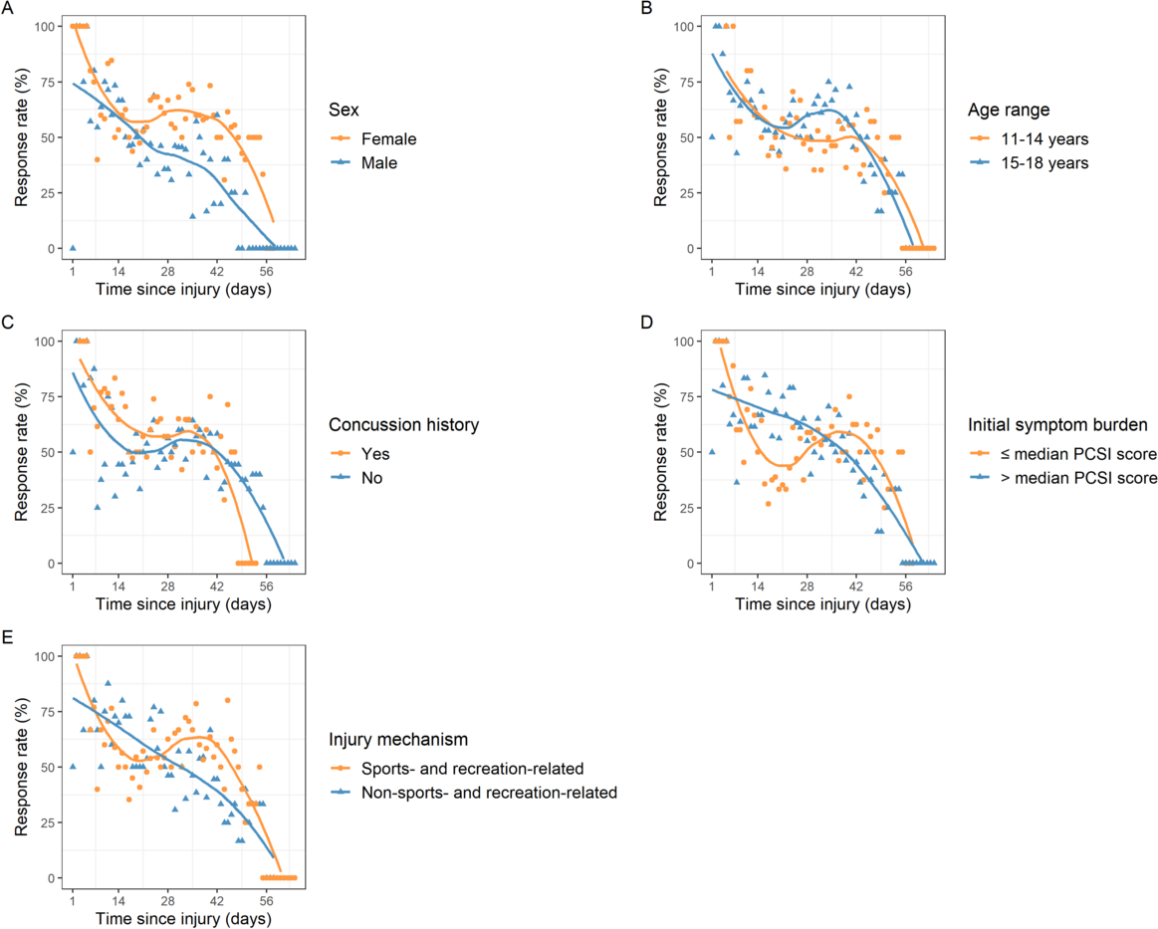
Comparisons of daily response rate by days since injury by (A) sex, (B) age group, (C) concussion history, (D) initial symptom burden, and (E) injury mechanism. The solid lines represent smoothed trend lines through local regression. Daily response was defined as a participant completing a response at least once per day (morning, afternoon, or evening). Response rate was defined as the number of participants who completed prompts divided by the number of participants who received notifications for that day. PCSI: Post-Concussion Symptom Inventory.

Figure 4 and Table S1 in Multimedia Appendix 1 show comparisons of response rate patterns by days since injury for demographic- and injury-related groups. Plateau periods were observed approximately 3-6 weeks post injury. Some of the subgroup patterns for days since injury followed those reported for days since app use. Evening response rates (median 37.0%, IQR 26.3%-46.0%) were significantly higher than morning (median 21.7%, IQR 12.8%-30.1%; *P*<.001) or afternoon response rates (median 28.1%, IQR 16.4%-35.1%; *P*=.003). Session and daily response rates in female patients with concussion were significantly higher than those in male patients with concussion (eg, median daily response rate 58.1%, IQR 50.0%-67.7% vs median 40.0%, IQR 0.0%-55.2%; *P*<.001). Participants with a history of at least one prior concussion had significantly higher session and daily response rates than those without a history of concussion (eg, median daily response rate 57.1%, IQR 50.0%-70.3% vs 45.5%, IQR 37.2%-58.3%; *P*=.003). Effects of injury mechanism and initial symptom burden were no longer significant when considering days since injury (*P*=.07-.88), and age group began to show an effect, with older teens demonstrating a slightly higher daily response rate than younger teens (median 54.4%, IQR 43.0%-66.7% vs median 50.0%, IQR 35.6%-57.1%; *P*=.03).

## Discussion

The purpose of this study was to investigate the self-reporting of symptom behaviors via an mHealth app (ReCoUPS) among adolescents with a concussion. Generally, response rate patterns varied between and within participants and days. Approximately half of the study sample responded at least once per day, with the evening representing the session time with the highest engagement across the enrollment period. Multiple demographic- and injury-related characteristics were associated with higher daily response rates, including female participants, longer time from injury to initiating mHealth monitoring, and history of a previous concussion. While not significant when considering daily response rates, there were session time differences in response rate by initial symptom burden and injury caused by sports and recreation activities. Responses by age did not show significant differences.

As expected, response rates started high, with nearly 90% of patients responding at least once on the first day of app use, then declined over time. Participants were not given reminders aside from the scheduled “pings” to complete a symptom evaluation. A prior study using a similar version of this app explored different incentive strategies and demonstrated how features like incentives that are more engaging and motivating for youth can increase response rates [27]. The decreasing engagement may reflect concussion recovery, as symptom presence and severity decreased over time in the studies using mHealth technology to monitor patients with a concussion [17,21,28,29]. Regarding response patterns across the three daily time points used in this study, evening (ie, prompts were sent at 8 PM and remained open until midnight) had the highest response rate and may be the optimal time to prompt adolescents in assessing their concussion symptoms. As evening hours may be less busy from school and extracurricular commitments, adolescents may prefer spending time in other activities using electronic devices (eg, smartphones and tablets), increasing the chance to respond to mHealth prompts.

Our findings reiterate the importance of sex differences in concussion. Female adolescents showed significantly higher response rates than male adolescents over recovery duration—for both time since injury and time since first app use. Factors attributing to response differences may be related to the manifestation of sex-based differences in symptoms that stem from differences in anatomy, biological underpinnings, and pubertal stage [6,30,31]. Behavioral expression and socially constructed roles associated with gender may also explain the higher rates in female adolescents [32]. For example, female adolescents have been shown to be more willing to report symptoms after concussion [33] and with greater frequency [6]. Particularly in digital use, female adolescents were found to have stronger motives for interpersonal communication and self-expression than male adolescents [34,35], which may explain why higher response rates were observed in female adolescents while using the mHealth app in this study.

Clear reporting differences existed between adolescents with and without concussion history. Adolescents with a concussion history had nearly twice the morning and afternoon response rates than those with no concussion history. For trends over time, similar patterns were observed. Studies have shown that adolescents with a history of a previous concussion continue to endorse more physical, cognitive, and fatigue symptom domains [36,37], and have longer recovery [8,38] than those without a concussion history. Persistent symptom presence and severity in adolescents with previous concussions may drive them to monitor their symptom patterns more carefully and frequently than those without a concussion history. Additionally, adolescents with a concussion history may have established trustworthy relationships with clinical providers during previous health care visits and, thus, may be more likely to follow assigned mHealth instructions such as reporting daily symptoms.

Adolescents with sports- and recreation-related injury demonstrated slightly higher response rates than those with non–sports- and recreation-related injury, particularly for the afternoon and evening session times. The desire to return to play may primarily motivate adolescents with a concussion to adhere to the instructions from health care providers with the hope of shortening recovery time [39,40]. Especially for adolescent athletes, there is a concern that absence from games or practice can let teammates down or even lead to their replacement on the team [41,42]. Individual desire to maintain team and peer acceptance in sports, and social support may drive adolescents who are injured via sports-related mechanisms to actively engage in mHealth solutions.

The differences in response rate between higher-than-median symptom burden versus lower-than-median symptom burden were inconsistent. For example, evening and daily response rates in participants with higher symptom burden were greater; however, morning response rates showed the opposite trend. High symptom burden may affect self-reporting behaviors both positively and negatively [28]. Adolescents with a concussion and higher symptom burden may demonstrate a stronger motive to engage with medical care compared to those with a lower symptom burden, facilitating mHealth app use to report daily symptoms. Alternatively, electronic devices (eg, computers, mobile phones, and tablets) may induce concussion-related symptoms such as headache and increased sensitivity to light [43,44], which could prevent adolescents with a concussion from adhering to digital health services or interventions. Our finding warrants further study to investigate symptom burden–related barriers that prevent self-reporting to mHealth services.

Reporting behavior was also affected by the time between injury and initial engagement with the app. Our finding showed that adolescents whose first use of the mHealth app was more than 7 days post injury demonstrated greater response rates than those whose first use was within 7 days of injury. Our finding may imply that the adolescents whose symptoms are sustained or even increased after 7 days post injury may have stronger motives in engaging in processes to support their recovery. Early presentation for medical care has been found to lower the risk of prolonged recovery [7,38,45]; therefore, those who present later and thus engage with the app later may be motivated to find ways to relieve symptom burden and may view regular engagement with the app as one means to do so. Encouraging reporting in the early period may be needed to help adolescents and families understand their symptom trajectory as well as how their health care provider can use that information to determine the next steps.

Older adolescents (ages 15-18 y) demonstrated slightly greater daily response rates than younger adolescents (ages 11-14 y), although most differences were not significant. Although it has been suggested that younger adolescents may be more likely to self-report concussions due to less negative perceptions of reporting injuries [46,47], ecological momentary assessment research has suggested that young children may find self-report methods more challenging to engage in [48]. Additionally, a larger foundation of concussion knowledge may guide older adolescents to report symptoms more regularly; previous research has suggested an association between age and concussion knowledge [46,49,50]. The younger participants may be less informed about concussion knowledge and therefore less likely to report.

There were several limitations in this study. First, the sample size in this study was small, though similar to or greater than other mHealth studies on patients with concussion. These data provide guidance for considerations for the implementation of mHealth in future studies of youths with a concussion with larger sample sizes. Second, our study enrolled adolescents aged 11-18 years, but participants were predominantly older adolescents (ages 15-18 years). Thus, the results should not be extrapolated to the younger pediatric population. Third, participants were predominantly White. This limitation warrants further study to investigate the application of mHealth technology in diverse communities, which may help clinicians and researchers understand the barriers to digital health equity, especially for non-Hispanic Black communities [9]. Fourth, because concussion cases were from a specialty care referral program, reporting behaviors may be biased toward adolescents who seek specialist care, which may include a population with more prolonged recovery. Lastly, our response rates were lower than some existing literature [23,29,48]; however, this was likely due to the lack of incentive strategies, lack of reminder prompts, and longer tracking duration compared to those studies. Dynamic incentivization showed higher response rates (IQR 47.6%-82.5%) in youths with concussion from the emergency department setting when tracking their daily symptoms compared with flat incentivization (IQR 20.6%-68.3%) [27].

In summary, adolescents with concussion demonstrated the ability to regularly report concussion symptoms via an mHealth tool in their natural living environment without financial compensation. To optimize future mHealth tool use from both the research and clinical perspectives, and for adolescents with concussion, evening may be the best prompt time. Response rates among adolescents varied by certain demographic- and injury-related characteristics. Multiple groups were more likely to engage in reporting daily symptoms: female patients, those who had a longer time from injury to app use, and those with a history of prior concussions. Using mHealth apps to document symptom presence and intensity daily can improve the reliability of self-reported data on symptom history at regular clinic visit intervals by eliminating the reliance on memory. Accurate and reliable measurement of postconcussion symptoms on a more granular basis than is captured during clinical visits could further improve clinical decision-making for personalized treatment. This is particularly important for a concussion, which is a dynamic traumatic brain injury in which symptom burden can increase or decrease rapidly. To promote adolescents’ adherence to mHealth use, especially for those groups who had low engagement in the monitoring app studied herein, future studies may improve mHealth app features like adding reminder prompts and using incentive strategies or adaptive digital concussion assessments to increase response rates.

## Supplementary material

10.2196/53186 Multimedia Appendix 1 Supplementary equations, figure, and table.

## Abbreviations

mHealth: mobile health
PCSI: Post-Concussion Symptom Inventory
ReCoUPS: Recovering Concussion Update on the Progression of Symptoms
