# Supplementary material for: Response Rate Patterns in Adolescents With Concussion Using Mobile Health and Remote Patient Monitoring: Observational Study
Source: JMIR Pediatr Parent. 2024 May 6;7:e53186. doi: 10.2196/53186 (PMC11089889; doi:10.2196/53186)
Supplement: Multimedia Appendix 1 [file pediatrics-v7-e53186-s001.docx]

**Supplementary Material**

**Supplementary Equations**

Response rate (RR) by session time and daily for each day, where day is examined two ways: by days since first app usage and by days since injury.

$$\begin{aligned} Time-based RR by session time=\frac{\sum participants who completed responses for each session time on that day}{\sum participants who received prompts for each session time on that day} \#\left( 1 \right) \end{aligned}$$

$$\begin{aligned} Time-based Daily RR =\frac{\sum participants who completely responded at least once on that day}{\sum participants who received prompts on that day} \#\left( 2 \right) \end{aligned}$$

**Figure S1.** Number of participants who received and responded to prompts in the morning, afternoon, evening, and daily by days since injury


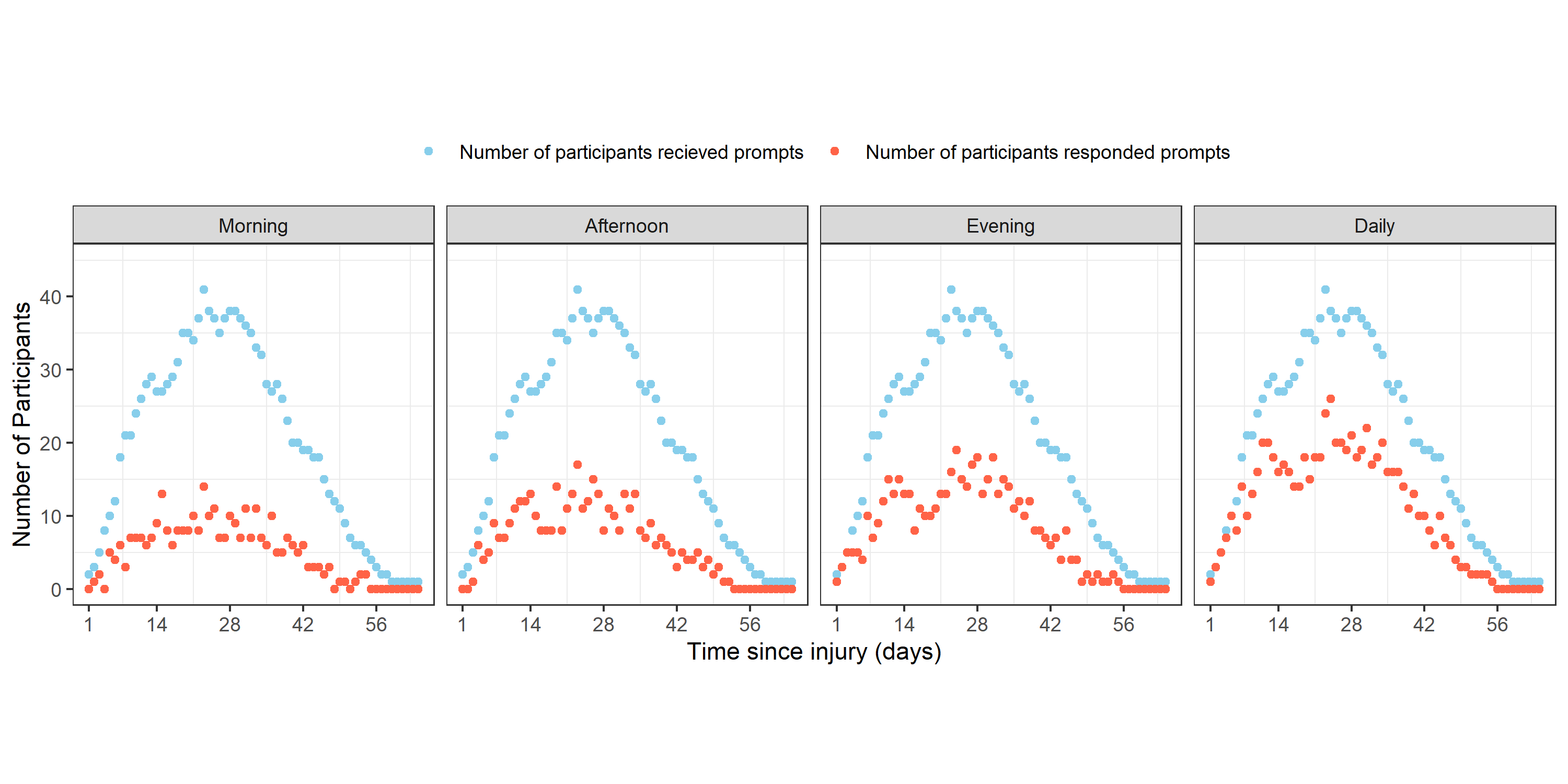


**Table S1.** Response rate by day since injury overall and across time-, demographic-, and injury-related features.

| **Variables** | **Morning (%)** | ***P*** | **Afternoon (%)** | ***P*** | **Evening (%)** | ***P*** | **Daily (%)** | ***P*** |
| --- | --- | --- | --- | --- | --- | --- | --- | --- |
|  | **Median (Q1, Q3)** |  | **Median (Q1, Q3)** |  | **Median (Q1, Q3)** |  | **Median (Q1, Q3)** |  |
| **Overall** | 21.7 (12.8, 30.1)) |  | 28.1 (16.4, 35.1) |  | 37.0 (26.3, 46.0) |  | 51.4 (33.3, 59.7) |  |
| **Sex** |  |  |  |  |  |  |  |  |
| Male | 14.3 (0.0, 27.1) | **< .001** | 20.0 (0.0, 31.8) | **.01** | 20.0 (0.0, 35.9) | **< .001** | 40.0 (0.0, 55.2) | **< .001** |
| Female | 25.0 (20.0, 33.3) |  | 28.6 (23.1, 37.3) |  | 46.2 (35.9, 51.6) |  | 58.1 (50.0, 67.7) |  |
| **Age range** |  |  |  |  |  |  |  |  |
| 11-14 years | 22.2 (0.0, 28.0) | .15 | 29.0 (14.3, 42.9) | .29 | 35.3 (24.3, 41.6) | .06 | 50.0 (35.6, 57.1) | **.03** |
| 15-18 years | 25.0 (11.1, 33.3) |  | 29.3 (16.7, 36.4) |  | 40.0 (30.1, 47.1) |  | 54.4 (43.0, 66.7) |  |
| **Injury mechanism** | |  |  |  |  |  |  |  |
| SSR injury | 25.0 (8.3, 36.4) | .14 | 33.3 (19.5, 38.5) | .21 | 40.0 (27.9, 50.0) | .14 | 54.2 (40.0, 65.9) | .76 |
| Non-SSR injury | 16.0 (0.0, 33.3) |  | 25.0 (8.5, 39.6) |  | 33.3 (17.5, 45.5) |  | 50.0 (33.3, 66.7) |  |
| **Concussion history** | |  |  |  |  |  |  |  |
| Yes | 25.0 (15.6, 32.7) | **.04** | 33.3 (22.3, 47.3) | **< .001** | 42.9 (32.6, 52.7) | **<** .**01** | 57.1 (50.0, 70.3) | **<** .**01** |
| No | 16.7 (7.9, 28.9) |  | 24.0 (9.6, 31.2) |  | 33.3 (22.2, 42.9) |  | 45.5 (37.2, 58.3) |  |
| **Initial symptom burden** | |  |  |  |  |  |  |  |
| > median score (47) | 21.1 (6.6, 30.8) | .16 | 25.0 (13.8, 37.0) | .07 | 37.9 (22.3, 50.0) | .88 | 54.2 (33.3, 66.7) | .81 |
| ≤ median score (47) | 23.8 (12.5, 35.7) |  | 31.2 (18.8, 42.9) |  | 37.5 (25.0, 50.0) |  | 53.3 (37.5, 62.5) |  |

Note: Q1 denotes the first quartile; and Q3 denotes the third quartile; SRR denotes sports and recreation-related.
